# Intersecting Paths to Health: A Factor Analysis Approach to Socioeconomic and Environmental Determinants in Indiana

**DOI:** 10.3390/ijerph22020219

**Published:** 2025-02-04

**Authors:** Siavash Ghorbany, Ming Hu, Siyuan Yao, Chaoli Wang, Matthew Sisk, Quynh C. Nguyen, Kai Zhang

**Affiliations:** 1 Department of Civil and Environmental Engineering and Earth Sciences, College of Engineering, University of Notre Dame, Notre Dame, IN 46556, USA; sghorban@nd.edu; 2 School of Architecture, University of Notre Dame, Notre Dame, IN 46556, USA; 3 Department of Computer Science, College of Engineering, University of Notre Dame, Notre Dame, IN 46556, USA; syao2@nd.edu (S.Y.); chaoli.wang@nd.edu (C.W.); 4 Lucy Family Institute for Data & Society, University of Notre Dame, Notre Dame, IN 46556, USA; msisk1@nd.edu; 5 National Institute of Nursing Research, Bethesda, MD 20892, USA; 6 Department of Population and Community Health, College of Public Health, The University of Texas Health Science Center at Fort Worth, Fort Worth, TX 76107, USA; kai.zhang@unthsc.edu

**Keywords:** public health, health equity, health burdens, socioeconomic disparities, chronic health conditions

## Abstract

Public health is the basis of society’s well-being and the nation’s development. Despite the importance of this factor and huge investments in the health sector in the United States, public health is facing enormous challenges due to the unknown nature of the influential variables in this sector. This research aims to investigate the influential variables on public health from different sources including the demographic features, built environment, socioeconomic variables, and environmental factors impact on 30 major health issues. To achieve this goal, this study utilizes exploratory factor analysis and multiple regression methods on the data obtained from the state of Indiana. The results indicated that health issues and influential factors can be divided into five main factors. This study identifies Health Burdens and Socioeconomic Disparities as a key factor, encompassing a wide range of health issues and socioeconomic variables, highlighting a significant association between socioeconomic disparities, poor health outcomes, and environmental exposures. The analysis underscores the intricate relationship between socioeconomic status, health behaviors, chronic diseases, and environmental factors, suggesting that effective interventions must address healthcare access, quality, and broader determinants of health to improve outcomes in affected communities. The results of this study can be helpful to public health policymakers, urban planners, and future public health researchers.

## 1. Introduction

The status of public health is a crucial determinant of a nation’s development and its ability to effectively harness human resource potential. However, the United States presents a paradoxical scenario in the realm of public health outcomes. According to the Centers for Disease Control and Prevention, the U.S. ranks unfavorably among 38 affluent nations concerning various critical health indices, registering the lowest life expectancy as well as the highest newborn and maternal mortality [1]. Additionally, the nation reports disproportionately high incidences of deaths attributable to diseases that are typically preventable or treatable, and it exhibits the highest suicide rate [1,2].

The incongruity is further underscored by the healthcare expenditure patterns. The U.S. allocates a significant proportion of its Gross Domestic Product (GDP) to healthcare on a per capita basis, surpassing other high-income nations [2]. Despite such investment, the U.S. remains the sole country among its peers without a system of universal healthcare coverage [1]. This discrepancy extends to healthcare access and utilization; Americans tend to have fewer physician consultations and the country has one of the lowest densities of practicing physicians and hospital beds per 1000 people. Paradoxically, the U.S. leads in rates of influenza and breast cancer screening but trails in the uptake of COVID-19 vaccinations [1,2].

Extensive research has established that a substantial proportion of health-related issues can be attributed to environmental determinants and living condition [3,4]. Factors influencing population health include characteristics of the built environment such as neighborhood proximity, urban heat islands, building materials, and exposure lead exposure [5,6]. Additionally, environmental changes encompassing shifts in climate change, extreme weather events, and air pollution [5], as well as demographic variables like age demographics, genetic predispositions, and the impact of systematic racism, etc.) [7,8,9], play a role. Moreover, the intersection of public health with social justice, including energy affordability and implications of poverty, further complicates the landscape of public health [7,10].

While these factors are often studied individually, understanding their interplay and combined impact on public health outcomes requires further empirical investigation, particularly in specific geographic contexts. Vulnerable populations frequently face double exposure, where environmental risks—such as poor air quality or proximity to hazardous sites—intersect with socioeconomic disadvantages, such as poverty and lack of access to healthcare [11,12,13]. An intersectional approach highlights how structural inequalities, environmental hazards, and individual health behaviors compound to worsen public health.

Although prior studies have examined the independent effects of these variables, few have systematically addressed their combined, multi-level impact. For example, the interplay between energy burdens, environmental hazards, and demographic vulnerabilities highlights the urgent need for interdisciplinary approaches to health equity research. The complex web of these factors suggests that localized studies can yield more nuanced insights into their collective influence on community health. It is the interaction of diverse elements, rather than isolated factors, that must be scrutinized to elucidate the complex dynamics affecting public health.

This study adopts a holistic methodological approach by integrating individual, multi-level, and environmental data to assess health disparities in Indiana. Investigating the concept of double exposure provides a novel framework for understanding how intersecting variables affect health outcomes.

To address this problem, this research aims to investigate the underlying factors that are associated with 30 major health problems in the United States in the state of Indiana. This study presents a pioneering effort that integrates consideration of health issues, demographic factors, built environment characteristics, environmental conditions, energy consumption, and socioeconomic status to evaluate health challenges in the United States. Utilizing explanatory factor analysis, the study has three primary objectives: (1) categorize the factors impacting the health problems and rate them based on their importance; (2) interpret the health problems correlation and underlying causes; and (3) develop a model to predict the general health status in census tracts, including mental health, physical health, and general health. A combination of exploratory factor analysis and regression models has been used to execute at the census tract level within Indiana. The paper is structured into Sections of literature review, methodology and materials, results and discussion, and conclusions.

## 2. Background and Existing Studies

The U.S. Department of Health and Human Services introduces economic stability, educational access, healthcare access, built environment, and social and community context as the social determinants of health [14]. Other researchers have introduced the built environment as the structural determinant of health [6]. The results of this study showed that the built environment characteristics such as greenness, single-family or multi-family nature of the buildings, and street direction are associated with health problems such as obesity, diabetes, drinking problems, and mental health [6]. Another study showed that more minor features of the built environment such as visible utility wires can impact the tendency to physical activities and result in obesity, diabetes, and other health problems [15]. Phan et al. (2020) achieved similar results and showed the presence of crosswalks can be associated with obesity and cardiovascular problems [16]. Other researchers emphasized the role of green spaces in the built environment on mental health status [17].

Environmental factors play a vital role in human health as well. In 2015, over 9 million premature deaths were reported as a result of pollution [18]. The studies show that environmental factors such as air pollution are a significant risk for chronic non-communicable diseases, especially cardiovascular health [19]. Chronic kidney disease is another health problem associated with these factors [20]. Other studies emphasized the importance of environmental factors such as exposure to toxic substances and infectious elements on the health problems in North America [21]. It has been stated that these factors not only might cause health problems directly but also could activate certain genes in the human body that result in critical health problems [21]. However, a lack of studies on measuring the ranked importance of these factors on the different health problems can be seen, and recent research has proposed more concentration on the relationship between environmental factors such as air pollution and physical health problems [22].

Demographic variables, on the other hand, have a crucial role in public health, specifically mental health, which also impacts the overall immune system reliability [23,24,25]. Socio-economic factors, on the other hand, impact the human lifestyle, nutrition, and necessities of life that can cause a diverse range of physical and mental health problems [26,27]. Sameroff and Seifer (2021) also indicate that poverty is one of the factors that influence children’s mental health status [28].

While most of the previous studies have mainly focused on a one-dimensional perspective toward health problems, including investigating one factor or one group’s impact on health problems, only a few researchers have worked on extracting the influential factors. Che and Shi (2020) worked on assessing the urban health level using several environmental and social factors. This study focused on GPD and consumption level as socio-economic factors, solid waste, wastewater, soot emission, and forestry as environmental ones, as well as several life factors, including population, access to libraries, state insurance, and traffic accidents [29]. However, this study used factor analysis to find the similarity and rank the cities’ health status based on these factors.

While the impact of external variables on health outcomes is broadly acknowledged, the comprehensive integration of these factors within an interdisciplinary framework remains underdeveloped in existing literature. The establishment of a descriptive framework that systematically categorizes these determinants and discerns their relative influence is crucial. Such a tool not only advances academic research but also informs evidence-based policymaking, particularly in fostering health equity at an urban level.

## 3. Methods and Materials

### 3.1. Research Flowchart

**Figure 1:**
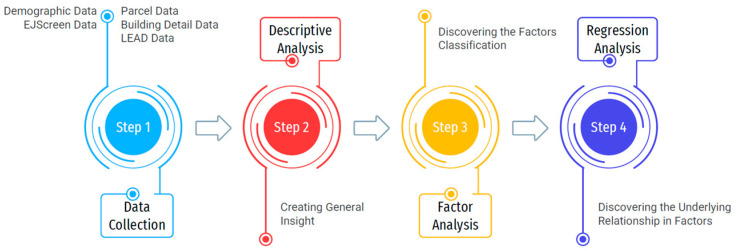
The research methodology flowchart.

As demonstrated in Figure 1, the research methodology of this study is divided into four main stages. Stage one, data collection, including collecting and merging different data to create a unified dataset for the state of Indiana on a census tract scale, including environmental, demographic, health, building attributes, and energy information. Afterward, the descriptive analysis was implemented in stage two to provide a general insight into the data characteristics and build the initial hypotheses. Next, a factor analysis was employed to determine the variable classifications and describe the health issues categorized with other parameters. Lastly, the ordinary least square (OLS) regression and several machine learning methods were deployed to discover the underlying intensity of the influential factor and provide the most suitable model for health issues prediction in each census tract. The details of each stage are explained in the following Sections.

### 3.2. Data Collection

The data collected in this research covered health problems, demographics, property, energy usage, and environmental data. Five different datasets were deployed to achieve this. Table 1 shows the list of variables extracted from each dataset.

#### 3.2.1. Health Issues Data

The extraction of the disease rate in each census tract as the dependent variable in this study was the first step of this research. To accomplish this, the data from the Centers for Disease Control and Prevention (CDC) were used [30]. The data were retrieved from PLACES: Local Data for Better Health. The CDC Foundation, the Robert Wood Johnson Foundation, and the CDC collaborated on PLACES. Across the nation, PLACES offers health data for tiny communities. This helps local health departments and jurisdictions plan public health initiatives by helping them better understand the spatial distribution and burden of health measures in their communities, independent of population size or rurality. Thirty different health issues in the census tract scale were extracted from this dataset (see Table 1).

#### 3.2.2. Demographic Data

Demographic data include different ethnicities, gender-related, and age-group information that can impact the health status in each region. The 2019 five-year aggregated American Community Survey (ACS) data were used to gather the variables in this category on the census tract scale [31]. ACS delivers annual data on U.S. social, economic, and housing characteristics, aiding community planning. Its 5-year estimates provide reliable data for small areas and groups, covering over 578,000 geographic areas across 87 summary levels without a population threshold [31]. The variables extracted from this dataset can be seen in Table 1.

#### 3.2.3. Property Data

The data related to the properties in Indiana were released in 2019 [35]. These data include different building attributes including the heating systems of the properties, the envelope materials, and the structural materials of the buildings. The parcel number for each property was assigned to the corresponding census tract using ArcGIS and then the data were grouped based on the census tract FIPS code. FIPS code is a unique identifier associated with each census tract. To complete the property-related data, authors previous research [32,36,37] was used to extract the window-to-wall ratio data from Google Street View (GSV) images using a Convolutional Neural Network (CNN) model.

#### 3.2.4. Energy Data

Average energy burden, defined as the energy expenditure ratio to household income, is another variable that represents the household’s well-being and its residents’ characteristics at the same time. Therefore, the next step was to extract and calculate the average energy burden in the census tract scale from the Low-Income Energy Affordability Data (LEAD) Tool [33].

#### 3.2.5. Environmental Data

Environmental factors, including air quality, buildings’ proximity to certain facilities, and exposure to certain elements, can cause health problems. Therefore, the environmental factors were the other set of variables that were added to the dataset. To accomplish this, the Environmental Justice Screening and Mapping Tool (EJScreen) data from the United States Environmental Protection Agency (EPA) were used [34]. Overall, 28 variables were extracted from this dataset (see Table 1) and the total number of variables being used in this study reached 76. All the extracted covariates were matched based on the census tract. The next step was to analyze the integrated dataset to gain an insight into the used variables and their statistical attributes. The process of executing this analysis is explained in the following Sections.

#### 3.2.6. Data Integration

This study utilized a combination of secondary data aggregated at the census tract level from multiple publicly available sources and primary data for the property data. The integration of these datasets allowed for an interdisciplinary analysis of demographic, socioeconomic, built environment, energy, and environmental factors and their impacts on public health. As explained in each Section, the extracted data for most of the variables are in the census tract scale. Therefore, the Federal Information Processing Standards (FIPS) code was used to integrate these datasets into a single dataset containing all the variables in Table 1. This includes EJScreen environmental data, LEAD energy data, ACS demographic data, and CDC health issues data. For the property data (extracted from the CNN model on GSV data), the coordination of Google panorama images was used to aggregate the data into the census tract level and then combine that with the rest of the datasets.

### 3.3. Descriptive Data Analysis

After creating the census tracts dataset, the data were cleaned, and missing values were omitted. The finalized data included about 80% of all census tracts in Indiana out of 1511 tracts in this state. Since these tracts were distributed uniformly throughout the state, the data analysis outcome will not be impacted much. Afterward, the descriptive analysis was performed to extract the mean, median, range, and quartiles of the data to provide insight into the distribution of the variable and check for any anomalies in the data. Following the validation of the data, Pearson Correlation was implemented to provide a general perspective into the data and check for the influential factors to develop the initial results hypotheses and understand the internal relationship among the covariates. Following the Pearson Correlation, the Variance Inflation Factor (VIF) was calculated for the variables to address the multicollinearity issue that can impact the interpretability of the linear regression models [32]. The numbers 5 or 10 are usually used as the cut-off for the VIF method [32]. This study used the number 5 as the threshold to consider even more conservative measures for its reliability.

### 3.4. Factor Analysis

#### 3.4.1. Data Suitability for Factor Analysis

Exploratory factor analysis (EFA) is a statistical method used to identify underlying variables, or factors, that explain the pattern of correlations within a set of observed variables [38,39]. It is used to reduce the number of variables in a dataset by finding the underlying structure, helping in data simplification and interpretation, and categorizing the variables with the same behavior in one class [40]. Exploratory factor analysis aids in hypothesis testing and the development of new theories by revealing the relationships among observed variables [41]. This technique is commonly applied in psychology, marketing, finance, and other fields to uncover hidden dimensions of complex concepts.

Considering the relatively huge number of variables in this study and the need to reveal the underlying structure of the covariates, factor analysis was used. The first step in determining the suitability of the factor analysis is the ratio of variables to the data observations [41]. As a rule of thumb, the ratio between 20:1 to 5:1 is acceptable for the factor analysis, and this study consisted of 1175 observations (census tracts) with 76 variables, making an approximate 15:1 ratio, which is suitable for factor analysis [42]. The next step is to prove the data’s suitability through statistical tests. Bartlett’s test of sphericity and Kaiser–Meyer–Olkin (KMO) are commonly used preliminary tests to assess the suitability of data for factor analysis and have been implemented in this study to check whether the correlations between items are sufficient for conducting exploratory factor analysis [43]. Bartlett’s Test of Sphericity evaluates the hypothesis that the correlation matrix is an identity matrix where a significant test result (*p*-value < 0.05) suggests that there is sufficient correlation between the variables to proceed with factor analysis.

The KMO measure assesses the adequacy of sampling by comparing the magnitude of observed correlation coefficients to the magnitude of partial correlation coefficients. KMO ranges from 0 to 1, with higher values indicating that factor analysis may be appropriate. A commonly used rule of thumb is that a KMO value greater than 0.6 is considered acceptable, values above 0.8 are considered good, and values below 0.5 suggest that factor analysis may not be suitable [43].

#### 3.4.2. Determining the Number of Factors

After assuring about the suitability of the data, the number of factors for an EFA analysis should be determined [44]. To implement this, two common methods, including choosing the eigenvalues larger than one and a Scree plot, are used [45]. Since the KMO in this study was high and, accordingly, the number of factors with larger than 1 eigenvalue was unreasonably high, the Scree plot method was used to determine the number of factors. In this method, the elbow point on the scree plot can be chosen as the desired number of factors [45].

#### 3.4.3. Calculate the Loading Factors and Corresponding Variables

The loading factors for each variable were extracted along with five factors, as the number selected through the scree plot method. Afterward, the variables with a loading factor larger than 0.5 were assigned to the corresponding factor [46]. Whenever values above 0.5 were observed in two different factors, the factor with a larger loading factor was selected for the variable. This threshold ensures a meaningful and strong relationship between variables and factors, consistent with widely accepted practices in exploratory factor analysis [46,47]. Following extracting the five selected factors, Cronbach’s alpha (α) was used to examine the internal data consistency (reliability) following the identification of possible causes [48]. Three alphas were produced to evaluate the data consistency and reliability under factors one to five since Cronbach’s alpha is frequently used to assess how closely linked the set of variables discovered under the underlying factors are as a group [49]. Moreover, the KMO score for each of the factors was calculated to validate the factors. After extracting and validating the factors, the underlying structure of the variables can be interpreted.

### 3.5. Prediction Models

#### 3.5.1. Ordinary Least Square (OLS) Regression

Ordinary Least Square (OLS) is a highly interpretable model that can reveal the interaction of the variables and how they are impacting the predictor variable. The dependent variables in this study were the health problems and specifically the mental health and physical health status in the census tracts. Therefore, the OLS was deployed to extract the influential covariates on health issues listed in Equation 1. To determine the coefficients of the OLS model, Equation (1) is solved where X is the matrix of the independent variables, and Y is the vector of target variables.

(1) $$\hat{\beta }={\left(X^{T}X\right)}^{-1}X^{T}Y$$

Since $\hat{\beta }$ is an estimated coefficient of the OLS regression model with uncertainty, the *p*-value is estimated and *p*-values below 0.05 are considered significant and reject the null hypothesis for the variable, meaning it is influencing the dependent variable. Equation 2 estimates the *p*-values of the OLS model where $\beta_{h}$ is the hypothesized value (in here zero), and $\hat{\beta_{j}}$ is the estimated coefficient, and SE determines the standard error in this of the $\hat{\beta_{j}}$ [50].

(2) $$t=\frac{\hat{\beta_{j}}-\beta_{h}}{\mathrm{SE}\left(\hat{\beta_{j}}\right)}$$

#### 3.5.2. Machine Learning Regression Models

After implementing the OLS, the influential variables and their impact significance are determined. Although OLS provides the most interpretable model and can be a necessary step for selecting variables rationally, it does not always provide the most accurate prediction model. Therefore, this research examined the machine learning methods support vector regression (SVR), decision tree regression, random forest regression, and XGBoost regression models to assess the possibility of developing a model with higher accuracy. Except for decision trees, all these models are black box models, and consequently, their prediction power costs a loss of interpretability in the analysis. The machine learning models were run through a grid search and were tested through a 5-fold cross-validation for finding the best hyperparameters and validating their results [32,51,52,53,54].

To assess the performance of the machine learning models and compare them to the OLS model, the Root Mean Square Error (RMSE), Mean Square Error (MSE), Mean Absolute Error (MAE), and R^2^ were in this research [55,56]. Equations (3)–(6) demonstrate the formula of these measures where the predicted value is shown as $y_{i}$, $y_{i}^{p}$ represents the estimated data, and ${\bar{y}}^{p}$ indicates the mean value. In these equations n is the sample size, RSS is the sum of squares of residuals and TSS is the total sum of squares.

(3) $$R^{2}=1-\frac{RSS}{TSS}$$

(4) $$RMSE=\;\sqrt{\frac{\sum_{i=1}^{n}{\left(y_{i}-y_{i}^{p}\right)}^{2}}{n}}$$

(5) $$MSE\;=\;\frac{1}{n}\sum_{i=1}^{n}{\left(y_{i}^{p}-y_{i}\right)}^{2}$$

(6) $$MAE=\frac{1}{n}\sum_{i=1}^{n}\left|y_{i}^{p}-y_{i}\right|$$

## 4. Results

### 4.1. Descriptive Data Analysis Results

**Figure 2:**
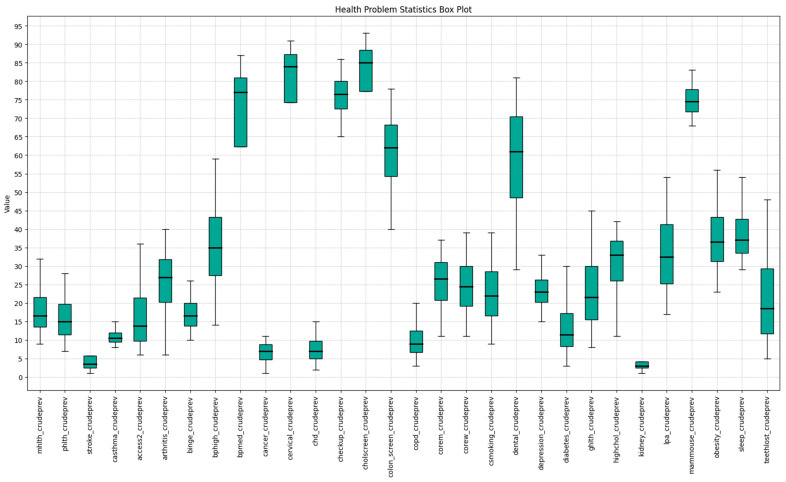
The Health Problems Boxplots.

In our analysis of Indiana’s health metrics, detailed in Figure 2, we found the crude prevalence of cholesterol screening among adults aged ≥ 18 (cholscreen_crudeprev) leading statewide, with a peak prevalence of 93%. The crude prevalence of cervical cancer screening among adult women aged 21–65 (cervical_crudeprev) was a close second, reaching a high of 91%. At the lower end, the crude prevalence of chronic kidney disease among adults aged ≥ 18 (kidney_crudeprev) and the crude prevalence of stroke among adults aged ≥ 18 (stroke_crudeprev) was observed with minimum prevalence rates near 1%. Cancer, excluding skin cancer, and coronary heart disease presented higher mean rates, averaging 7%, compared to the lower prevalence rates of kidney disease and stroke.

Significant disparities in health behaviors were evident across census tracts. For example, the crude prevalence of visits to a dentist or dental clinic among adults aged ≥18 (dental_crudeprev) ranged from 34% to 81%, indicating the highest variability. Similarly, notable variations were observed in the crude prevalence of all teeth lost among adults aged ≥ 65 (teethlost_crudeprev) and the crude prevalence of high blood pressure among adults aged ≥ 18 (bphigh_crudeprev), with ranges spanning 5–48% and 14–59%, respectively.

The demographic factors were the next set of variables examined in this research. The general statistics of these variables are represented in Figure 2. As shown in Table 2, the highest mean value for demographic group distribution belongs to the white population in Indiana. On average, about 82% of the census tract populations are identified as white population, while these values also have the largest standard deviation and range from no white population in some census tracts to 100% white population in other ones.

Asian and Hispanic populations had the lowest average among all demographic groups with 1.49 and 7.26 percent, respectively. These demographic groups also had the lowest standard deviations as well with about 3.43 for the Asian population and 9.78 for the Hispanic ones. There was almost a balanced distribution among the male and female population in the state of Indiana. Regarding the age groups, the 65- to 74-year-old population had the largest proportion of the elderly group with 9.58% while the over 85-year-old group only covers about 2.17% of the whole over 65-year-old population. Overall, on average, about 16.38% of the population in the Indiana census tracts are over 65 years old while this number reaches above 78% in some census tracts.

**Figure 3:**
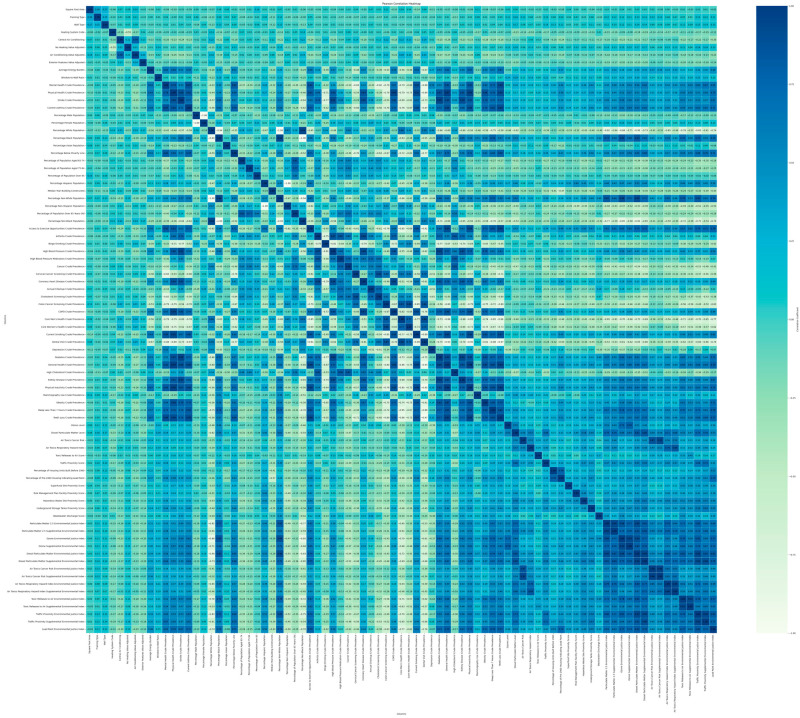
Pearson Correlation among the variables.

After analyzing the general statistics, the Pearson Correlation was deployed to generate a general insight into the variable’s relationship (see Figure 3). The bottom right and bottom left variables in this graph are mostly related to the EJScreen dataset, which represents the environmental factors. Among these variables, some health problems, including mental health, physical health, stroke rate, and asthma, showed a significant positive correlation with the environmental factors while some other diseases demonstrated a negative or non-significant relationship. Table 3 shows the full list of health problems correlated with nature and environmental problems.

Our analysis revealed a notable trend where the predominantly non-Hispanic have a negative correlation with the environmental factors. This suggests that as environmental problems escalate, there is a tendency for the Hispanic and Black populations in that area to increase. Additionally, factors such as average energy burden and window-to-wall ratio (WWR) displayed a positive correlation with the environmental stressors. Moreover, a rise in the poverty percentage was linked with an increase in environmental risks. Interestingly, within the senior demographic, the 65–74 age cohort exhibited the strongest inverse relationship with environmental risk factors.

### 4.2. Factor Analysis Results and Interpretation

#### 4.2.1. Correlation Analysis and Data Suitability

The correlation analysis shown in Figure 3 shows that almost all the variables have some internal relationship with one or more of the other variables. Moreover, the ratio of the data is about 15:1, which puts it in the acceptable range for exploratory factor analysis [42]. To proceed with this verification in a statistical way, Bartlett’s Test of Sphericity and the KMO test were deployed. Bartlett’s Test of Sphericity indicated a chi-square value of approximately 206,059 and a *p*-value of 0. This proved adequate intercorrelations between the variables. Furthermore, the overall KMO result was equal to 0.937. The closeness of this value to one indicated that the selected set of variables is good for factor analysis. To indicate the number of factors, the scree plot and the Elbow Method were used, which resulted in the fifth eigenvalue as the elbow point [45]. Therefore, the number of factors was set to five.

#### 4.2.2. Exploratory Factor Analysis Results

After determining the overall suitability and number of factors, the exploratory factor analysis was implemented. The KMO was calculated for each of the five factors and Cronbach alpha was also calculated to check the data reliability (see Table 4). The variables with loading factors larger than 0.5 were placed in the corresponding factor. In contrast, whenever a variable was mutual between two factors, the larger factor loading was considered the main category for the variable. Therefore, a total of 60 variables were assigned to the factors and 16 variables were removed.

The omitted variables were mostly from the property data, including the heating equipment, the square footage, framing, and WWR. From the demographic data, the population of females was removed, and RESI_AIR and PWDIS variables from the environmental category were omitted. Also, the complementary variables of the demographic data were omitted to keep the dataset matrix invertible for factor analysis (e.g., the male populated were removed, and the female was kept since they are complementary). As demonstrated in Table 4, all the factors had a KMO larger than 0.7, showing strong associations of variables with this factor. However, Cronbach’s alpha for Factor 3 and Factor 4 is insignificant. Therefore, the first two factors, alongside factor 5, are the most reliable ones due to the high KMO and Alpha values at the same time.

Factor one, named “Health Burdens and Socioeconomic Disparities” encompasses 22 health-related variables and 2 socioeconomic variables: average energy burden and below poverty level population percentage. Notably, the variable “access2_crudeprev”, denoting the lack of health insurance, intersects both health and socioeconomic factors. Additionally, this category includes environmental variables, specifically the EJ index for toxic air releases and lead paint exposure, highlighting the nexus between environmental hazards and socioeconomic conditions.

High factor loadings for the crude prevalence of physical health (0.948), adults’ mental health (0.849), and fair or poor health among adults (0.935) reveal the critical impact of socioeconomic factors on health. The correlation here underscores a pervasive link between poverty and deteriorating physical and mental well-being. The adverse effects of environmental factors, such as lead paint and toxic air, are evident in various health detriments, including fatigue, memory loss, increased blood pressure, headaches, dizziness, elevated blood pressure, and impaired motor abilities. [57,58]. Adults might suffer damage from lead paint exposure at even low levels since the lead paint chips are not the only thing that poison; dust and particles containing lead, which can be released from deteriorating paint or during renovation activities, also pose a significant risk when they are inhaled or ingested. Although the building age is not in this category, Table 4 shows that there is a −0.43 correlation between the lead paint and building age, which is aligned with the EPA manifest about lead paint [59]. In addition to the general health indicators, the inclusion of variables like the prevalence of diagnosed diabetes (0.792), high blood pressure (0.703), coronary heart disease (0.716), chronic obstructive pulmonary disease (0.868), and obesity (0.871) underscores the burden of chronic diseases within communities facing socioeconomic challenges. Contrastingly, preventive measures such as cancer screenings and dental visits are negatively loaded, suggesting that with rising socioeconomic disparities, the uptake of health-maintaining practice diminishes.

Overall, this factor captures a complex interplay between health status, behaviors, and socioeconomic and environmental conditions. It suggests that individuals and communities facing higher socioeconomic disparities are also likely to experience a range of health burdens, including higher rates of chronic diseases, poorer mental and physical health outcomes, and lower engagement in preventive health behaviors. The inclusion of environmental exposures further broadens the scope of these disparities, linking them to the physical environment in which people live. The strong association between these variables under Factor 1 indicates that interventions aimed at improving health outcomes in these communities need to be multifaceted, addressing not only healthcare access and quality but also broader socioeconomic and environmental determinants of health.

Factor 2 was labeled as “Aging and Chronic Health Conditions”, considering the variables in this category. This factor had a KMO of 0.778 and an alpha value of 86.53, which suggests a relatively good level of adequacy for factor analysis, indicating that the variables within this factor are well-correlated and suitable for analysis as a group. Of the 30 health problems, 20% were related to this factor. The factor includes variables such as the percentage of the population aged 65–74 years (0.557), 75–84 years (0.591), and over 65 years (0.755), indicating a significant focus on the older adult segment of the population. The loadings suggest that as the proportion of older adults in the population increases, the connection between aging and chronic health conditions increases as well.

The crude prevalence of arthritis (0.822) and the prevalence of taking medicine for high blood pressure control (0.892) have high factor loadings, emphasizing the commonality of these conditions among older adults and the importance of medication management in this population. Medication management system for older adults has proven to be an important criterion at the society well-being level, which is a challenge in many societies [60,61,62], and therefore considering these specific health problems with underlying association with the elderly might help toward better medication management.

The prevalence of cancer, excluding skin cancer, is another high loading variable in this factor. With a loading of 0.868, cancer is being highlighted as a significant health concern for the aging population, reflecting both the incidence and the challenges associated with cancer management and care in older adults. Moreover, cardiovascular problems seem to play a vital role in the elderly. The inclusion of variables related to cholesterol screening (0.662) and the prevalence of high cholesterol (0.849) suggests a focus on preventive health measures and the management of cardiovascular risk factors. The routine checkup prevalence (0.675) further supports the importance of regular healthcare engagement for early detection and management of chronic conditions. Overall, this factor can be considered as a factor needing lots of management and executive decisions to support the public health status at older ages.

Factor 5, “Environmental Health Risks”, another important factor in this study, discusses the air toxicity variables as one of the categories. This category does not combine with any of the health problems directly. However, as seen in Figure 3, these variables are correlated with most of the health problems and “Toxic Releases to Air EJ Index” in factor one. Therefore, it can be interpreted that these are the most crucial air toxicity indexes that cause an overall unhealthy environment for other health problems. The overall interpretation of these factors shows that socioeconomic conditions, public health management with the aim of elderly well-being, and environmental factors play the most significant role in society’s health status.

### 4.3. Multiple Regression Model Results

**Figure A1:**
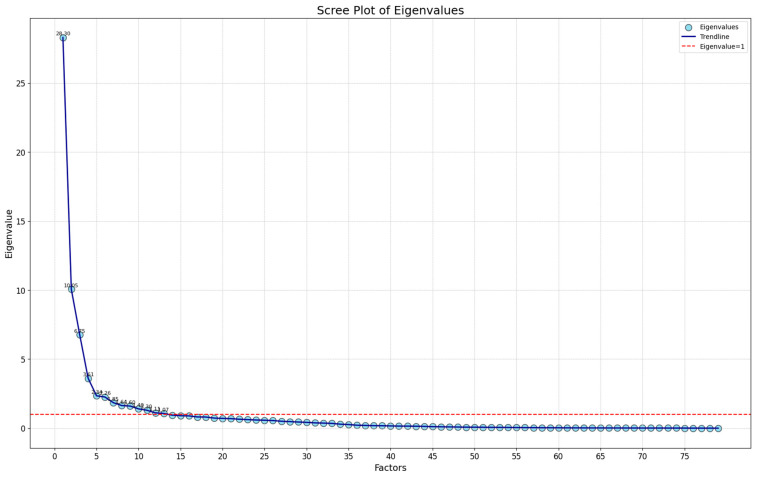
The Eigenvalues plot for Factor Analysis.

After analyzing the different factors, the mental health problem and the physical health problem were chosen as the representatives of Factor 1 and were health problems that had two of the highest loading factors and referred to two different states of health problems. The multiple regression model was run on all the health problem models (find the full regression results in the Figure A1), and the insignificant variables were removed. The remaining variables produced an R^2^ of 0.82 for the mental health problem (see Table 5) and 0.767 for the physical health problem (see Table 6).

The F-statistics of the model were 441 and 319 for the mental health regression model and physical health regression model, respectively, determining that the overall models’ results are statistically significant. A threshold of 0.05 was chosen to select the variables in the models. As demonstrated in Table 5, the mental health model ended up with Average Energy Burden and Poverty and the socioeconomic factors, the percentage of Asian, Black, and over 65 years population as the demographic factors, and seven environmental variables (see Table 5).

**Figure 4:**
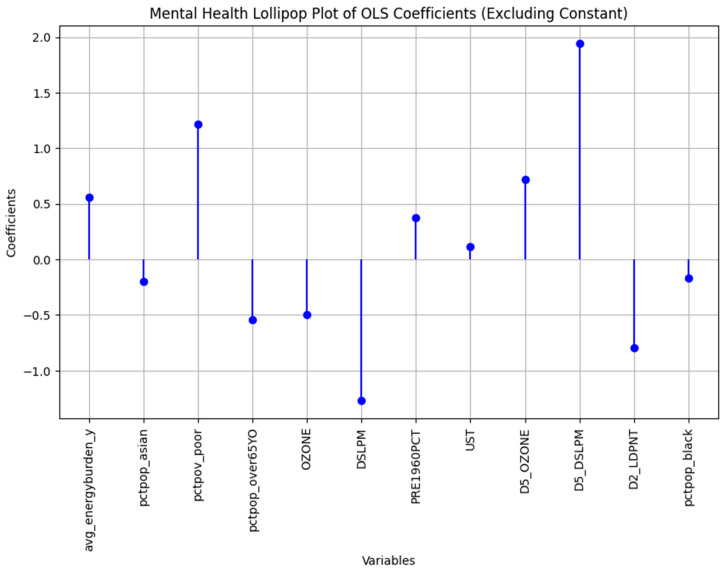
The mental health regression standardized coefficients comparison.

**Figure 5:**
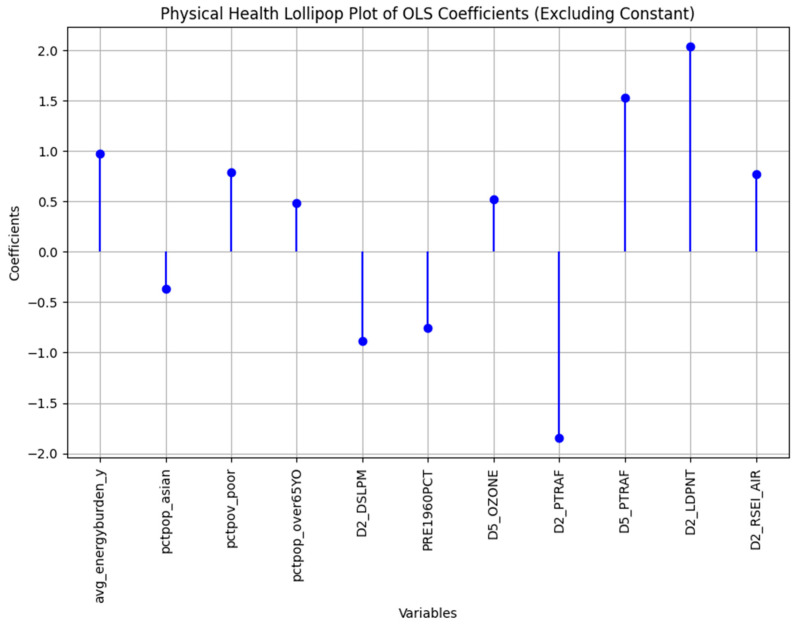
The physical health regression standardized coefficients comparison.

The demographic and socioeconomic variables were the same in both mental health and physical health problems. However, the influential environmental factors were different. The physical health problems model had Traffic proximity EJ Index (D2_PTRAF), Traffic proximity Supplemental Index (D5_PTRAF), and Toxic Releases to Air EJ Index (D2_RSEI_AIR) while the mental health model included the Underground storage tanks (UST) as the built environment environmental factor. Therefore, while the air toxicity and traffic-related indicators in the urban environment impact physical health, it seems the mental health problems are triggered by the compounds from underground tanks that store petroleum or hazardous substances [63]. Figure 4 and Figure 5 show the comparison between different factors that influence mental health and physical health status, respectively.

Moreover, the Average Energy Burden is one of the mutual parameters between both health problems that have the highest coefficient among the variables ranging from 0 to 100 percent. That means the average energy burden is the most influential factor among the socioeconomic parameters presented in this study. For mental health problems, a 1% increase in the average energy burden on average increases the mental health problem by 0.38% while increasing the physical health problems by an even greater influence, 0.66%. However, it should be noted that the coefficients and their sign cannot be interpreted accurately in this model since the variables are highly correlated, and a change in one of them changes the other ones significantly (see Figure 3). For instance, the value of the lead paint is negative in Table 5, denoting an association between an increase in lead paint and a decrease in mental health problems, which is not physically logical. This is due to the extremely high correlation between the lead paint index and other environmental variables.

That said, the R^2^ and R^2^ adjusted values suggest that both the presented models in Table 5 and Table 6 are good fits for predicting physical and mental health problems. Although it is not possible to interpret the values’ exact influence on the dependent variables, they are still statistically significant and are associated with the mentioned health problems.

## 5. Discussion

This study provides a comprehensive investigation into the determinants of public health in Indiana, utilizing exploratory factor analysis (EFA) and multiple regression models to identify the interplay between socioeconomic, demographic, environmental, and health variables. The findings highlight five distinct factors, with “Health Burdens and Socioeconomic Disparities” emerging as the most significant. This factor underscores the intertwined impact of socioeconomic inequities, chronic diseases, and environmental exposures, such as lead paint and toxic air releases, on community health. These results emphasize the necessity of multifaceted interventions that address healthcare access, socioeconomic disparities, and environmental justice.

The identification of “Aging and Chronic Health Conditions” as a key factor reveals the heightened vulnerability of older adults to chronic illnesses like arthritis, high blood pressure, and cancer. This finding highlights the critical need for preventive healthcare measures and improved chronic disease management for aging populations. The strong correlations between aging demographics and these health conditions call for targeted public health policies to address the unique needs of older adults, including access to routine screenings and medication management systems.

“Environmental Health Risks” as a standalone factor reinforces the importance of addressing air toxicity and other environmental hazards. While these variables were not directly associated with specific health conditions in the factor analysis, their strong correlations with health outcomes indicate their indirect but significant influence on public health. This aligns with existing literature on the disproportionate environmental burden faced by vulnerable populations, emphasizing the need for stringent policies to mitigate exposure to air pollution and other environmental stressors.

The regression analyses further solidify the role of socioeconomic and environmental factors in shaping health outcomes. Variables like average energy burden and below-poverty population percentage showed strong associations with both mental and physical health problems, underscoring the pervasive impact of economic inequities on health. Interestingly, environmental variables such as traffic proximity and underground storage tanks were differentially associated with physical and mental health, respectively, suggesting nuanced pathways through which environmental exposures affect health.

Overall, this study highlights the multifactorial nature of public health determinants, with significant implications for policymakers, urban planners, and public health practitioners. Addressing health disparities requires an integrated approach that considers the synergistic effects of socioeconomic, environmental, and demographic factors. Future research should explore causal relationships and extend the analysis to other geographic areas for broader applicability.

## 6. Conclusions

This study explores the relationship between various demographic, housing, energy, and environmental factors on thirty key health issues within the state of Indiana. A comprehensive dataset was curated for this analysis. The research methodology used the exploratory factor analysis technique to extract the underlying structure of the variables and check the internal relationship between the data. The high internal correlation between the variables in this study made the factor analysis one of the most suitable models to investigate the underlying structure of the data.

The data were divided into five factors. The “Health Burdens and Socioeconomic Disparities” factor delineates the intertwining of health issues with socioeconomic and environmental factors, indicating the communities facing greater socioeconomic challenges also experience heightened health risks, including mental, physical, and chronic diseases, with significant environmental impacts such as lead exposure and air toxicity.

Factor 2 correlates aging with chronic conditions like arthritis and high blood pressure, emphasizing the importance of preventive care and medical management for the elderly. This informs healthcare professionals and policymakers of the necessity for tailored services, such as increased access to screenings and chronic condition management, geared toward an aging demographic. While Factor 5 included the environmental group of the variables and although a direct health problem was not presented in this factor, the high correlation between these factors and air toxicity presented in Factor 1 suggests a clear need for policies and interventions aimed at reducing emissions of toxic air pollutants and mitigating their impacts on vulnerable populations. It underscores the importance of environmental justice in public health efforts, recognizing that some communities may bear a disproportionate burden of these health risks due to factors beyond their individual control, such as location, socioeconomic status, and access to healthcare.

The findings of this study can be useful to public health researchers and policymakers, environmental agencies, and energy departments. Further studies are suggested to concentrate on extracting the cause and result relationship of the variables and expanding the current research to more diverse areas to increase the generalizability of the research.

While this study provides valuable insights, several limitations warrant consideration. First, the method used in this study is a correlational statistical method and should not be interpreted as a causal relationship. Furthermore, the use of secondary data aggregated at the census tract level may obscure individual-level variations. Second, the cross-sectional nature of the analysis limits the ability to infer temporal dynamics or long-term trends. Future research should explore longitudinal data to better understand the evolution of health disparities over time. Additionally, expanding the study to include other states or regions would enhance the generalizability of the findings.

By addressing these limitations and building on the insights provided, future studies can further elucidate the complex interplay of factors influencing public health and inform more effective interventions to promote health equity and well-being. Deploying simulation tools can also help in inferring the results from a correlational perspective to a causal inference [64,65,66].

## Figures and Tables

**Table 1:** The variables from different datasets.

Dataset	Category	Number of Variables	Variables Used
[30]	Health Problems	30	access2_crudeprev, arthritis_crudeprev, binge_crudeprev, bphigh_crudeprev, bpmed_crudeprev, cancer_crudeprev, casthma_crudeprev, cervical_crudeprev, chd_crudeprev, checkup_crudeprev, cholscreen_crudeprev, colon_screen_crudeprev, copd_crudeprev, corem_crudeprev, cprew_crudeprev, csmoking_crudeprev, dental_crudeprev, depression_crudeprev, diabetes_crudeprev, ghlth_crudeprev, highchol_crudeprev, kidney_crudeprev, ppa_crudeprev, mammouse_crudeprev, mhlth_crudeprev, obesity_crudeprev, phlth_crudeprev, sleep_crudeprev, stroke_crudeprev, teethlost_crudeprev
[31]	Demographic Data	8	pctpop_female, pctpop_white, pctpop_black, Asian Population Percentage, Below Poverty Population Percentage, pctpop_hispanic, y_built_median, Population Over 65 Years Percentage
[32]	Property Data	9	SQUARE_FOOT_AREA, FRAMING_TYPE, WALL_TYPE, HEATING_SYSTEM_CODE, CENTRAL_AIR_CONDITIONING, NO_HEATING_VALUE_ADJ, AIR_CONDITIONING_VALUE_ADJ, EXTERIOR_FEATURES_VALUE_ADJ, WWR
[33]	Energy Data	1	Average Energy Burden
[34]	Environmental Data	28	OZONE: Ozone depletion DSLPM: Diesel particulate matter CANCER: Air toxics cancer risk RESP: Air toxics respiratory HI RSEI_AIR: Toxic Releases to Air PTRAF: Traffic proximity PRE1960: Housing units built before 1960 PRE1960PCT: Lead Paint PNPL: Superfund proximity PRMP RMP: facility proximity PTSDF: Hazardous waste proximity UST: Underground storage tanks PWDIS: Wastewater discharge D2_PM25: Particulate Matter 2.5 EJ Index D5_PM25: Particulate Matter 2.5 Supplemental Index D2_OZONE: Ozone EJ Index D5_OZONE: Ozone Supplemental Index D2_DSLPM: Diesel particulate matter EJ Index D5_DSLPM: Diesel particulate matter Supplemental Index D2_CANCER: Air toxics cancer risk EJ Index D5_CANCER: Air toxics cancer risk Supplemental Index D2_RESP: Air toxics respiratory HI EJ Index D5_RESP: Air toxics respiratory HI Supplemental Index D2_RSEI_AIR: Toxic Releases to Air EJ Index D5_RSEI_AIR: Toxic Releases to Air Supplemental Index D2_PTRAF: Traffic proximity EJ Index D5_PTRAF: Traffic proximity Supplemental Index D2_LDPNT: Lead paint EJ Index
**Total**		**76**	

**Table 2:** The demographic factors statistics.

Variables	Count	Mean	Std	Min	25%	50%	75%	Max
Male Population%	1176	49.22	4.68	29.42	46.69	49.27	51.53	100.00
Female Population%	1176	50.78	4.68	0.00	48.47	50.74	53.31	70.58
White Population%	1176	81.83	21.94	0.00	76.52	91.11	96.27	100.00
Black Population%	1176	10.53	18.87	0.00	0.34	2.14	10.78	97.48
Asian Population%	1176	1.49	3.43	0.00	0.00	0.32	1.49	38.86
Poor Population%	1175	15.64	11.68	0.18	7.49	12.23	20.71	79.06
Hispanic Population%	1176	7.26	9.78	0.00	1.52	3.84	8.85	76.84
Non-White Population%	1176	18.17	21.94	0.00	3.73	8.89	23.48	100.00
Non-Hispanic Population%	1176	92.74	9.78	23.16	91.15	96.16	98.48	100.00
Non-Black Population%	1176	89.47	18.87	2.52	89.22	97.87	99.66	100.00
65–74 y Population%	1176	9.58	3.65	0.00	7.06	9.34	11.67	30.90
75–84 y Population%	1176	4.63	2.52	0.00	2.85	4.33	5.99	36.84
Over 65 y Population%	1176	16.38	6.07	0.06	12.40	16.13	19.84	78.94
Over 85 y Population%	1176	2.17	1.95	0.00	0.94	1.70	2.86	21.05

**Table 3:** The health problems correlated with environmental factors.

Significant Positive Correlation	Significant Negative Correlation	Non-Significant Correlation
Mental Health Physical Health Stroke Asthma Health Insurance Access High Blood Pressure Coronary Heart Disease Chronic Obstructive Pulmonary Current Smoking Prevalence Diabetes Prevalence General Health Chronic Kidney Disease Physical Inactivity Mammogram Use Obesity Prevalence Insufficient Sleep Teeth Loss Prevalence	Binge Drinking Cancer (excluding skin cancer) Cervical Cancer Screening Cholesterol Screening Colorectal Cancer Screening Men’s Preventive Health Women’s Preventive Health Dental Visit Prevalence High Cholesterol Prevalence	Arthritis Prevalence High Blood Pressure Medication Use Annual Medical Checkup Prevalence Depression Prevalence

**Table 4:** Factor Analysis Results.

Principal Factor	Nature	Variables	Factor Loading	*α*
Overall KMO = 0.937				
Factor 1 (Health Burdens and Socioeconomic Disparities)				**78.13%**
KMO = 0.957	+	Average Energy Burden	0.633	
	+	crude prevalence of mental health not good for ≥14 days among adults aged ≥ 18	0.849	
	+	crude prevalence of physical health not good for ≥14 days among adults aged ≥ 18	0.948	
	+	crude prevalence of stroke among adults aged ≥ 18	0.783	
	+	Crude prevalence of current asthma among adults aged ≥ 18	0.870	
	+	Percentage of Population Below Poverty Level	0.720	
	+	crude prevalence of current lack of health insurance among adults aged 18–64	0.801	
	-	crude prevalence of binge drinking among adults aged ≥ 18	−0.653	
	+	crude prevalence of high blood pressure among adults aged ≥ 18	0.703	
	-	Crude prevalence of cervical cancer screening among adult women aged 21–65	−0.654	
	+	Crude prevalence of coronary heart disease among adults aged ≥ 18	0.716	
	-	crude prevalence of fecal occult blood test, sigmoidoscopy, or colonoscopy among adults aged 50–75	−0.844	
	+	crude prevalence of chronic obstructive pulmonary disease among adults aged ≥ 18	0.868	
	-	crude prevalence of older adult men aged ≥ 65 who are up to date on a core set of clinical preventive services: flu shot past year, PPV shot ever, Colorectal cancer screening	−0.815	
	-	crude prevalence of older adult men aged ≥ 65 who are up to date on a core set of clinical preventive services: flu shot past year, PPV shot ever, Colorectal cancer screening	−0.807	
	+	crude prevalence of current smoking among adults aged ≥ 18	0.925	
	-	crude prevalence of visits to dentist or dental clinic among adults aged ≥ 18	−0.922	
	+	crude prevalence of diagnosed diabetes among adults aged ≥ 18	0.792	
	+	crude prevalence of fair or poor health among adults aged ≥ 18	0.935	
	+	crude prevalence of chronic kidney disease among adults aged ≥ 18	0.737	
	+	crude prevalence of chronic kidney disease among adults aged ≥ 18	0.925	
	+	crude prevalence of obesity among adults aged ≥ 18	0.871	
	+	crude prevalence of sleeping less than 7 h among adults aged ≥ 18	0.806	
	+	crude prevalence of all teeth lost among adults aged ≥ 65	0.918	
	+	Toxic Releases to Air EJ Index	0.513	
	+	Lead paint EJ Index	0.661	
Factor 2 (Aging and Chronic Health Conditions)				**86.53%**
KMO = 0.778	+	Percentage 65–74 years Population	0.557	
	+	Percentage 75–84 years Population	0.591	
	+	Percentage over 65 years Population	0.755	
	+	Crude prevalence of arthritis among adults aged ≥ 18	0.822	
	+	crude prevalence of taking medicine for high blood pressure control among adults aged ≥ 18 years with high blood pressure	0.892	
	+	crude prevalence of cancer (excluding skin cancer) among adults aged ≥ 18	0.868	
	+	Crude prevalence of visits to doctor for routine checkup within the past year among adults aged ≥ 18	0.675	
	+	crude prevalence of cholesterol screening among adults aged ≥18	0.662	
	+	crude prevalence of high cholesterol among adults aged ≥ 18 who have been screened in the past 5 years	0.849	
Factor 3 (Environmental and Social Determinants)				53.44%
KMO = 0.773	+	Percentage of Hispanic Population	0.549	
	+	Diesel particulate matter	0.623	
	+	Traffic proximity	0.546	
	+	Housing units built before 1960	0.538	
	+	RMP facility proximity	0.536	
	+	Hazardous waste proximity	0.532	
	+	Underground storage tanks	0.535	
	+	Diesel particulate matter Supplemental Index	0.590	
	+	Traffic proximity EJ Index	0.577	
	+	Traffic proximity Supplemental Index	0.630	
Factor 4 (Demographic and Environmental Health Disparities)				27.89%
KMO = 0.794				
	-	Percentage of white Population	−0.748	
	+	Percentage of black Population	0.776	
	-	crude prevalence of depression among adults aged ≥ 18	−0.756	
	+	crude prevalence of mammography use among women aged 50–74	0.559	
	+	Ozone	0.503	
	+	Particulate Matter 2.5 EJ Index	0.568	
	+	Ozone EJ Index	0.677	
	+	Diesel particulate matter EJ Index	0.539	
Factor 5 (Environmental Health Risks)				**83.67%**
KMO = 0.729	+	Air toxics cancer risk	0.833	
	+	Air toxics respiratory HI	0.656	
	+	Particulate Matter 2.5 Supplemental Index	0.511	
	+	Air toxics cancer risk EJ Index	0.827	
	+	Air toxics cancer risk Supplemental Index	0.880	
	+	Air toxics respiratory HI EJ Index	0.675	
	+	Air toxics respiratory HI Supplemental Index	0.734	

**Table 5:** Mental Health Multiple Regression Results.

Prob > F = 0 F-Statistic: 441 No. Observations: 1175		Dep. Variable: Mental Health R-Squared: 0.820 Adj. R-Squared: 0.818 AIC: 3890 BIC: 3956				
Variables	Coefficient	Std. Error	t-Value	*p*-Value	[0.025	0.975]
Intercept	30.824	3.068	10.047	0.000	24.805	36.843
Average Energy Burden	0.377	0.032	11.696	0.000	0.314	0.441
Asian Population Percentage	−0.058	0.012	−4.890	0.000	−0.081	−0.034
Below Poverty Population Percentage	0.105	0.005	19.181	0.000	0.094	0.115
Population Over 65 Years Percentage	−0.089	0.007	−13.448	0.000	−0.102	−0.076
OZONE	−0.254	0.052	−4.849	0.000	−0.357	−0.151
DSLPM	−11.449	1.046	−10.945	0.000	−13.502	−9.397
PRE1960PCT	0.016	0.003	0.049	0.000	0.009	0.022
UST	0.026	0.010	2.497	0.013	0.006	0.047
D5_OZONE	0.119	0.024	4.938	0.000	0.072	0.166
D5_DSLPM	0.285	0.025	11.435	0.000	0.236	0.334
D2_LDPNT	−0.047	0.008	−6.151	0.000	−0.062	−0.032
pctpop_black	−0.009	0.004	−2.478	0.013	−0.016	−0.002

**Table 6:** Physical Health Multiple Regression Results.

Prob > F = 0 F-Statistic: 292.3 No. Observations: 1175			Dep. Variable: Physical Health R-Squared: 0.734 Adj. R-Squared: 0.732 AIC: 4590 BIC: 4651			
Variables	Coefficient	Std. Error	t-Value	*p*-Value	[0.025	0.975]
Intercept	9.118	0.255	35.726	0.000	8.617	9.618
Average Energy Burden	0.660	0.043	15.183	0.000	0.574	0.745
Asian Population Percentage	−0.107	0.016	−6.734	0.000	−0.138	−0.076
Below Poverty Population Percentage	0.068	0.007	9.485	0.000	0.054	0.082
Population Over 65 Years Percentage	0.079	0.009	8.903	0.000	0.062	0.097
D2_DSLPM	−0.049	0.010	−4.950	0.000	−0.069	−0.030
PRE1960PCT	−0.032	0.004	−7.333	0.000	−0.040	−0.023
D5_OZONE	0.087	0.015	5.880	0.000	0.058	0.116
D2_PTRAF	−0.109	0.015	−7.476	0.000	−0.137	−0.080
D5_PTRAF	0.225	0.024	9.182	0.000	0.177	0.273
D2_LDPNT	0.121	0.011	11.321	0.000	0.100	0.142
D2_RSEI_AIR	0.049	0.006	7.978	0.000	0.037	0.061

## Data Availability

The data that support the findings of this study are available upon request from the corresponding author, MH.

## Abbreviations

**Acronym**	**Description**	**Acronym**	**Description**
OZONE	Ozone Level	D2_PM25	Particulate Matter 2.5 Environmental Justice Index
DSLPM	Diesel Particulate Matter Level	D5_PM25	Particulate Matter 2.5 Supplemental Environmental Index
CANCER	Air Toxics Cancer Risk	D2_OZONE	Ozone Environmental Justice Index
RESP	Air Toxics Respiratory Hazard Index	D5_OZONE	Ozone Supplemental Environmental Index
RSEI_AIR	Toxic Releases to Air Score	D2_DSLPM	Diesel Particulate Matter Environmental Justice Index
PTRAF	Traffic Proximity Score	D5_DSLPM	Diesel Particulate Matter Supplemental Environmental Index
PRE1960	Percentage of Housing Units Built Before 1960	D2_CANCER	Air Toxics Cancer Risk Environmental Justice Index
PRE1960PCT	Percentage of Pre-1960 Housing Indicating Lead Paint	D5_CANCER	Air Toxics Cancer Risk Supplemental Environmental Index
PNPL	Superfund Site Proximity Score	D2_RESP	Air Toxics Respiratory Hazard Index Environmental Justice Index
PRMP	Risk Management Plan Facility Proximity Score	D5_RESP	Air Toxics Respiratory Hazard Index Supplemental Environmental Index
PTSDF	Hazardous Waste Site Proximity Score	D2_RSEI_AIR	Toxic Releases to Air Environmental Justice Index
UST	Underground Storage Tanks Proximity Score	D5_RSEI_AIR	Toxic Releases to Air Supplemental Environmental Index
PWDIS	Wastewater Discharge Score	D2_PTRAF	Traffic Proximity Environmental Justice Index
D2_LDPNT	Lead Paint Environmental Justice Index	D5_PTRAF	Traffic Proximity Supplemental Environmental Index
